# Investigate the Variations of the Head and Brain Response in a Rodent Head Impact Acceleration Model by Finite Element Modeling

**DOI:** 10.3389/fbioe.2020.00172

**Published:** 2020-03-18

**Authors:** Runzhou Zhou, Yan Li, John M. Cavanaugh, Liying Zhang

**Affiliations:** Department of Biomedical Engineering, Wayne State University, Detroit, MI, United States

**Keywords:** traumatic brain injury, head impact acceleration model, diffuse axonal injury, finite element rat model, brain strain, head linear acceleration, head angular velocity

## Abstract

Diffuse axonal injury (DAI) is a severe form of traumatic brain injury and often induced by blunt trauma. The closed head impact acceleration (IA) model is the most widely used rodent DAI model. However, this model results in large variations of injury severity. Recently, the impact device/system was modified to improve the consistency of the impact energy, but variations of the head kinematics and subsequent brain injuries were still observed. This study was aimed to utilize a Finite Element (FE) model of a rat head/body and simulation to investigate the potential biomechanical factors influencing the impact energy transfer to the head. A detailed FE rat head model containing detailed skull and brain anatomy was developed based on the MRI, microCT and atlas data. The model consists of over 722,000 elements, of which 310,000 are in the brain. The white matter structures consisting of highly aligned axonal fibers were simulated with transversely isotropic material. The rat body was modeled to provide a realistic boundary at the spine-medulla junction. Rodent experiments including dynamic cortical deformation, brain-skull displacement, and IA kinematics were simulated to validate the FE model. The model was then applied to simulate the rat IA experiments. Parametric studies were conducted to investigate the effect of the helmet inclination angles (0°–5°) and skull stiffness (varied 20%) on the resulting head kinematics and maximum principal strain in the brain. The inclination angle of the helmet at 5° could vary head linear acceleration by 8–31%. The change in head rotational velocity was inversely related to the change in linear acceleration. Varying skull stiffness resulted in changes in head linear acceleration by 3% but with no effect on rotational velocity. The brain strain in the corpus callosum was only affected by head rotation while the strain in the brainstem was influenced by the combined head kinematics, local skull deformation, and head-neck position. Validated FE models of rat impact head injury can assist in exploring various biomechanical factors influencing the head impact response and internal brain response. Identification of these variables may help explain the variability of injury severity observed among experiments and across different labs.

## Introduction

Traumatic Brain injury (TBI) is caused primarily by mechanical loading to the head. According to the report from the Centers for Disease Control and Prevention (Faul et al., 2010), an estimated 1.7 million TBIs occurred in the United States annually, of which 52,000 resulted in death, while 275,000 required hospitalization, with another six million individuals suffering neurobehavioral sequelae and functional loss. Diffuse axonal injury (DAI) is the most frequent type of TBIs in closed head injuries (Wright and Ramesh, 2012). It has been suggested that DAI be renamed to Traumatic Axonal Injury (TAI) due to the extent of the injury being multifocal among white matter structures rather than diffuse (Smith and Meaney, 2000). TAI is induced by rapid head acceleration/deceleration with a consequent tissue deformation leading to localized mechanical insult to the axons.

Researchers used various *in vivo* or *in vitro* animal models to introduce TAI in the laboratory settings to mimic human injury, and to investigate the underlying injury mechanisms. Compared to an *in vitro* model, *in vivo* models keep the complex extracellular environment and allow for tracking the complex physiologic, behavioral, and histopathological changes after a traumatic insult. The rodent is the most frequently used *in vivo* animal model in the brain injury research due to its low cost, small size, and standardization (Cernak, 2005; O’Connor et al., 2011; Xiong et al., 2013).

A challenge to the investigation of the closed head diffuse brain injury is the difficulty of inducing an isolated but significant degree of axonal injury without concomitant focal contusion and skull fracture. Marmarou’s impact acceleration (IA) model (Marmarou et al., 1994) is the most widely used rodent model to study TAI. This IA model can reliably produce different severities of axonal injury in a closed head impact injury. Briefly, the heads of the anesthetized animals are placed unrestrained in a prone position on a foam bed, adjusted to the end of the device, and a head impact is delivered via a free-falling weight. The drop weight and height can be adjusted to produce a graded axonal injury in the white matter (Foda and Marmarou, 1994; Marmarou et al., 1994; Beaumont et al., 1999; Kallakuri et al., 2003). Biomechanically, the IA model mimics a closed head injury, induced by a combined linear and angular head motion after impact. The injury was mainly diffuse with extensive traumatic axonal injury found in the discrete white matter tracts, including corpus callosum (cc) and brainstem (Marmarou et al., 1994). There are more than 1400 publications related to the use of Marmarou’s model in the last 25 years. Many aspects of TBI were studied by using this model, such as cellular and molecular response, histopathology of impaired axoplasmic transport (IAT) and neurofilament compaction (NFC), motor and cognitive deficits, oxidative stress and mitochondrial, and diagnose and treatment after TBI (Adelson et al., 1997, 2001; Heath and Vink, 1999; Schmidt et al., 2000; Stone et al., 2004; Macdonald et al., 2005; Fei et al., 2006; Marmarou and Povlishock, 2006; Kallakuri et al., 2007; Rafols et al., 2007; Vagnozzi et al., 2007; Sengul et al., 2008).

Despite the widespread utility of the Marmarou’s model in studying various aspects of TBI, there is very limited work on the understanding of the mechanical responses of the model (Gilchrist, 2004; Wang and Ma, 2010). In earlier kinematics studies of Marmarou’s model, only the impact velocity and supporting foam were studied to be the factors related to rat head kinematics which caused brain injury (Piper et al., 1996). The results showed the potential variations (impact velocity and supporting foam stiffness varied 40% and 53%) in the mechanical system, which may explain various mortality rates reported by different groups using the same model (Suehiro et al., 2001; Rhodes et al., 2002; Geeraerts et al., 2006; Marmarou et al., 2006; Ucar et al., 2006; Fei et al., 2007; Pascual et al., 2007).

Recently, the original model was modified in Wayne State University to eliminate the variation originated from impact velocity and supporting foam. The model has been expanded to monitor impactor velocity, head displacement into the foam, head linear kinematics and head angular kinematics at the impact (Li et al., 2011a, b). Results from this study for the first time offered the relationship between measured rat head kinematics, and the quantified axonal changes as well as biomarker change in both cerebrospinal fluid (CSF) and serum (Li et al., 2015). It was noted that even with improved consistency of the impact energy imparted to the rat head, some variabilities were still observed between the tests in terms of head kinematics (range of linear acceleration: 321–2313 g and rotation velocity: 58–181 rad/s of 2.25 m height drop cases), and the severity of the axonal change assessed by histopathology (range of TAI counting in cc: 23–913 and in py: 68–2417 of 2.25 m height drop cases). Noticeably, the quantified axonal injury severity varied between the left and the right hemispheres. Given that the impact was delivered to the center of the helmet at the midline of the skull, the role of the variability of boundary conditions surrounding the impactor surface, along with the helmet/head position during impact needs to be investigated to improve the consistence of the head responses to a given impact. It is hypothesized that the variation of the initial helmet angle with respect to the impactor surface during the impact and the rat skull stiffness may influence the energy transferring mode, and affects the translational and rotational motion of the rat head in response to the impact. Finite element modeling method is a useful tool for analyzing the effect of the various boundary conditions and simulating the physical phenomena. The objectives of this study were to develop a detailed rat head FE model to simulate the Marmarou’s IA experiments conducted recently, and to quantify the effect of initial conditions on the resulting brain tissue deformation pattern and severity, which may offer biomechanical basis for the understanding of the difference in TAI severity.

## Materials and Methods

### FE Model Development

#### Rat Head Model Development

**FIGURE 1:**
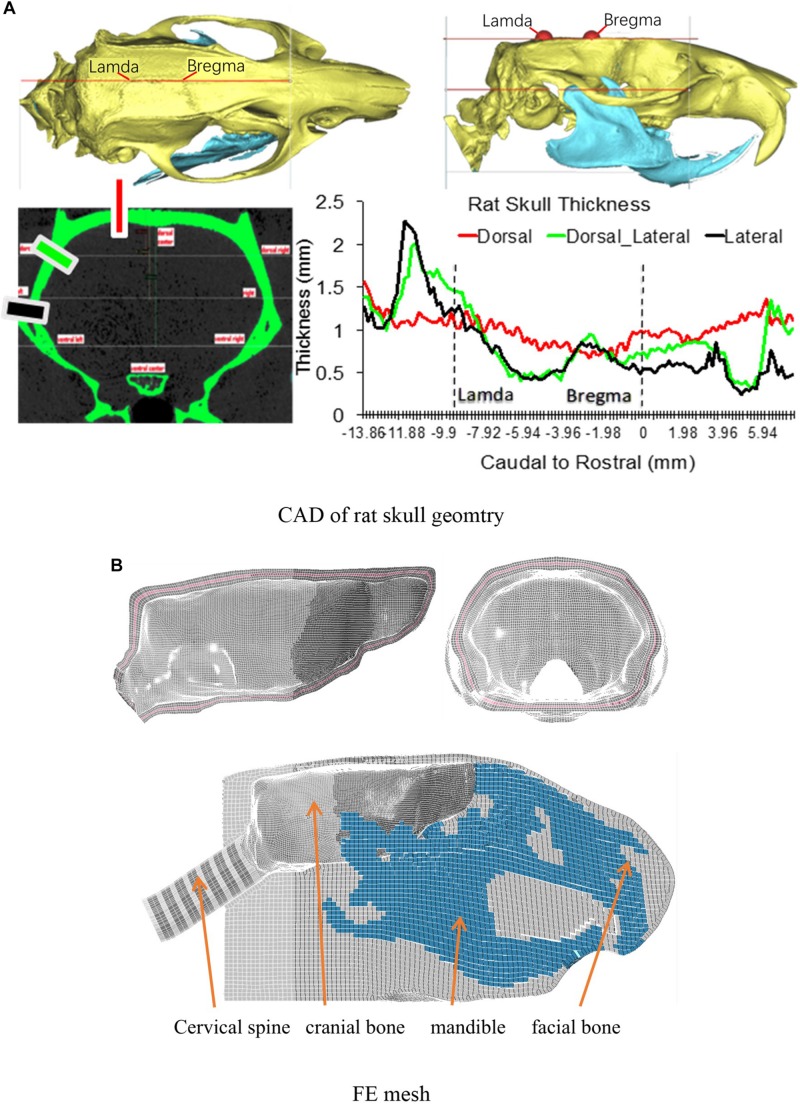
**(A)** The rat skull thickness variation measured from MicroCT scan data; **(B)** Rat skull mesh.

To develop the detailed FE model of the skull and the brain, the skull geometry of a 400 g Sprague Dawley (SD) rat was scanned by a MicroCT at 25 μm resolution. The brain geometry of the same rat was obtained from a series of MRI images (4.7 Tesla). The surface geometries of the skull and brain were then constructed using Mimics 11.11 (Materialise, Leuven, Belgium) based on the image data, and refined with reference to the rat brain atlas (Paxinos and Watson, 2007). The FE meshes of the skull and brain were then developed using a variety of FE mesh preprocessors, including Hypermesh (Altair Engineering, Troy, MI, United States), Morpher (DEP, Troy, MI, United States), and Hexmesher (DEP, Troy, MI, United States). To precisely represent the actual skull thickness in the FE skull model, the thickness profile from dorsal to ventral, rostral to caudal, were accurately meshed according to the MicroCT data (Figure 1A). The thickness of skull varied from one location to another. The lateral side from rostral to caudal had most variation ranging from 2.25 to 0.28 mm. The dorsal side had the least variation from 1.55 to 0.7 mm. Each of the inner and outer tables of the skull were meshed with two layers of elements and diploe was meshed with one-layer elements. The element size was segregated evenly into brick elements (Figure 1B) (average 0.2 mm). The skull meshed with five layers of the brick elements ensured proper simulation of the bending stiffness. The facial and mandible bones were meshed as one single component and constrained to the skull mesh. The facial soft tissue was meshed as one component and directly contacted with facial and mandible bone by common nodes. The facial bones and facial flesh were modeled with hexahedral elements with coarse resolution (0.6 mm) to save computational time.

**FIGURE 2:**
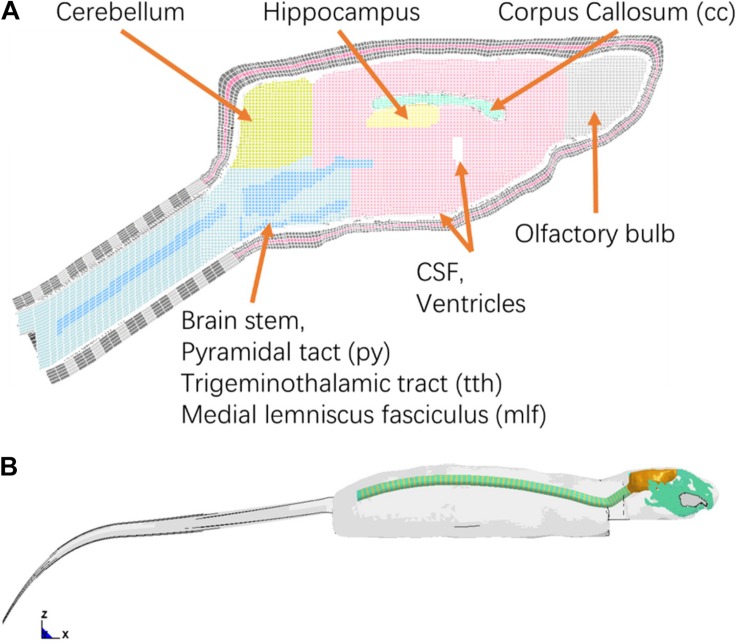
**(A)** Finite element mesh of the rat brain (Midline sagittal section view); **(B)** A lateral view of rat whole body FE model.

The intracranial components included the olfactory bulb, cerebral cortex, cc, hippocampus, cerebellum, ventricles, CSF, thalamus, brain stem, pia-arachnoid complex, and dura mater (Figure 2A). Several axonal fiber tracts in the brainstem including pyramidal tract (py), trigeminothalamic tract (tth), and medial lemniscus fasciculus (mlf), were segregated based on the brain atlas (Paxinos and Watson, 2007). All the anatomical structures of the brain were meshed with brick or hexahedral elements with element resolution between 100 and 200 microns. The element size meshed for the brain was approximately 0.2 mm × 0.2 mm. The element size chosen for the rat brain mesh was equivalent to element mesh size of the human head models (2 mm) (Wayne State University Head Injury Model 2001 and GHBMC head model) developed previously where the solution convergence was assured (Zhang et al., 2001; Mao et al., 2013). This element size ensures the scalability of the numerical results of the brain response between the FE models of the human, Rhesus monkey (Arora et al., 2019), and the current SD rat. The element size was sufficiently small to explicitly model the major fiber tracts (e.g., py, mlf, etc.) which were the key structures of axonal pathology that were quantified on an injury map at 0.2 × 0.2 mm resolution from our previous animal study (Li et al., 2011b). The total mass of the brain and head model was 2.3 and 42 g, respectively, confirming the values measured from a 400 g SD rat.

The vertebra and intervertebral disks for the neck were meshed with oval-shaped columns, with heights based on the MRI data (1.3 and 0.55 mm) for each vertebra segment. The mechanical properties of the vertebra and intervertebral disks were defined as elastic and viscoelastic materials respectively, based on reported literature data. The rat head-neck model consisted of over 0.83 million elements with a total mass of 76 grams.

#### Full Body Model Development

To simulate the physical rat head impact acceleration test, a whole-body FE model is needed in order to provide realistic physical boundary condition to the head/neck model. The geometry of the rat body was obtained from MRI (4.7 Tesla) images. The outer surface of body and the spine vertebrae were segmented using MIMICS. Instead of developing a detailed model for the rest of the body parts (Figure 2B), only the simplified spine structure and flesh were developed in the FE model. The model of the simplified spinal column consists of vertebral bodies and disks with cylindrical shape, where the diameters were close to the data from the MRI images. The rest of the body soft tissues (muscle and flesh) and organs were lumped into one component and meshed with tetrahedral elements. The overall material density was tuned to represent the mass of a 400 g SD rat within the range of mass of the SD rats used in the rat head impact tests (Figure 2B). The entire FE rat model was constructed with over 1.35 million elements. The element numbers of the brain, head, neck are 301, 724, and 119 k, respectively.

#### Material Models and Material Properties of Rat Model

All FE simulations were performed using a non-linear, dynamic, explicit FE solver LS-DYNA R6.1 (LSTC, CA, United States). The material properties assigned to each component of the rat head model were based on the published experimental data and the values used by the other FE head models (Shreiber et al., 1997; Gefen et al., 2003; Pena et al., 2005; Levchakov et al., 2006; Mao et al., 2006; Fijalkowski et al., 2009; Zhu et al., 2010; Wright and Ramesh, 2012; Lamy et al., 2013; Antona-Makoshi et al., 2014).

For the brain structures containing the gray matter, MAT_KELVIN-MAXWELL_VISCOELASTIC (MAT_061) was used to simulate shear behaviors under dynamic loading (Table 1). The materials were isotropic. The white matter in some of the brain regions contains highly organized axonal fibers, particularly in the cc, py, ml, mlf of the brainstem. Some experimental studies of brain material properties reported that white matters can be modeled as transversely isotropic in shear and compression (Arbogast and Margulies, 1998; Prange and Margulies, 2002; Ning et al., 2006; Hrapko et al., 2008; Van Dommelen et al., 2010). MAT_SOFT_TISSUE_VISCO material model (MAT_092) was used to model the transversely isotropic properties of the cc and various axonal fibers in the brainstem (Supplementary Appendix Table A1). The remaining white matter tissue without distinctive fiber directions was modeled as isotropic material with different shear property values from the gray matter (Table 1). All the brain tissues were assigned bulk modulus of 2 GPa due to the incompressibility of brain material.

**TABLE 1:** Material property.

Maxwell viscoelastic model				

Components	Short-term shear modulus (G_0_) (kPa)	Long-term shear modulus (G_i_) (kPa)	Beta (/ms)	References
Gray cortex	5.16	1.54	0.05	Zhang et al., 2001; Gefen et al., 2003
Hippocampus	10.32	3.08	0.05	
Cerebellum	4.64	1.38	0.05	
Pia-arachnoid	1379	153	0.04	Jin et al., 2007, 2011, 2014
Intervertebral disc	1000	100	0.05	Zhang et al., 2006; Kallemeyn et al., 2010

**Material property of the elastic parts**				

**Mat_ elastic**	**Elastic modulus (MPa)**		**Poisson’s ratio**	**References**

Dura mater	31.5		0.45	Mao et al., 2013
Skull, diploe layer	600		0.3	McElhaney et al., 1970; Motherway et al., 2009; Wood, 1971
Skull, cortical layer	15,000		0.3	
Vertebral body	7000		0.3	Kallemeyn et al., 2010

MAT_092 material uses Mooney-Rivlin hyper-elastic model to represent the isotropic matrix along with added fiber reinforce item; the viscosity was represented by using Prony series. The energy model of Mooney-Rivlin model with fiber reinforcement term is described by Eqs. 1 and 2 as follow:

(1) $$W=C_{1}\,\left({\tilde{I}}_{1}-3\right)+C_{2}\,\left({\tilde{I}}_{2}-3\right)+F\,\left(\lambda \right)+\frac{1}{2}\,K\,{\left[\ln\left(J\right)\right]}^{2}$$

(2) $$\frac{\partial F}{\partial \lambda }=\left\{\begin{array}{cc} 0\,\lambda <1\; & \\ \frac{C_{3}}{\lambda }[\exp\left(C_{4}\left(\lambda -1\right)-1\right]\lambda <\lambda^{*}\; & \\ \frac{1}{\lambda }\,\left(C_{5}\,\lambda +C_{6}\right)\,\lambda \geq \lambda^{*}\; & \end{array}\right\}$$

Where [$C_{1}\,\left({\tilde{I}}_{1}-3\right)+C_{2}\,\left({\tilde{I}}_{2}-3\right)$] represents the energy of isotropic hyper-elastic characteristic of matrix. [*F*(λ)] represents the fiber reinforced energy. [$\frac{1}{2}\,K\,{\left[\ln\left(J\right)\right]}^{2}$] is the energy caused by volume change. The derivative of [*F*(λ)] by [λ] is defined to capture the behavior of the crimped collagen (stress-strain curve). [λ] is the stretch point when the crimped fiber becomes straight. When [λ < λ^∗^], the two constants, *C*_3_ scales the exponential stresses and *C*_4_ is the rate collagen fiber uncrimping (Puso and Weiss, 1998). *C*_5_ represents the fiber reinforced stiffness, C_6_ is the continue point at [λ = λ^∗^]. The relaxation function used to represent the viscosity:

(3) $$G\,\left(t\right)=\sum_{i=1}^{6}S_{i}\,\exp\left(\frac{t}{T_{i}}\right)$$

S_*i*_ and T_*i*_ represented the portion of shear moduli and time characteristic. For simplification, only S_1_, S_2_ and T_1_, T_2_ were defined.

The matrix stiffness of the cc was based on shear modulus (Gi) of the gray matter, and matrix of the brainstem tracts was about two times stiff as cc. Their fiber direction enforcement was about two times as matrix when the fiber elongated (λ = 1.02). The axonal fiber tracts of brainstem (mlf, tth, and py) utilized the same material properties as the surrounding brainstem (Arbogast and Margulies, 1999; Prange et al., 2000; Prange and Margulies, 2002; Bain et al., 2003; Meaney, 2003; Ning et al., 2006; Karami et al., 2009). The assigned transversely isotropic materials allowed simulation of directional dependent peripteries of white matter fibers, as the tissue behaved two times stiffer for the elements stretching along fiber direction as compared to the other two directions. The skull, facial bones and vertebral bodies were defined as an elastic material and the values associated with them are shown in Table 1.

The rate dependent MAT_FU_CHANG_FOAM (MAT_083) was assigned to the supporting foam with four stress-strain curves at different loading rates (Supplementary Appendix Figure A3). These properties were obtained from foam compression tests and were validated previously by Zhang et al. (2011).

### Rat FE Model Validations

#### Validation 1: Dynamic Cortical Deformation

After creating the detailed FE rat head model, Dynamic Cortical Deformation (DCD) experiments reported by Shreiber et al. (1997) were simulated to validate the brain deformation computed by the FE model. According to the DCD experimental preparation, a 5 mm diameter craniotomy was performed over the left parietal and the dura was removed. Nine sets of vacuum pressure (2, 3, 4 psi with duration 25, 50, 100 ms) histories measured from the experiments were applied directly to the cortical surface via the pia-arachnoid membrane at the skull/dura opening site. The model predicted displacement at the cortical surface was compared to those measured experimentally.

To understand the effect of some biomechanical parameters on the model predicted displacement results in response to the suction force with varying peak pressure and duration, a parametric study was conducted. Firstly, since the material of the pia-arachnoid membrane was much stronger than the brain tissue (about 100 to 1000 as strong as brain tissue under tension and traction loading) (Jin et al., 2011), the type of the material model used to simulate pia-arachnoid membrane could affect the deformation magnitude and temporal response. Secondly, the characteristics of brain as it moves against the skull at the brain/skull interface in response to the suction force could influence the brain surface deformation pattern and magnitude. Different types of the brain-skull interface models were investigated. Table 2 shows the matrix with a total of nine simulations to determine the best combination of the interface and the material model that matches the FE model results to those of experimental results.

**TABLE 2:** Parametric study matrix for improving DCDvalidation results.

Variable 1: Various contact types to simulate brain/skullinterface	Variable 2: Various material models forpia-arachnoid membrane
Sliding w/o friction (S)	Elastic
Sliding tiedbreak (normal failure) (ST)	Elasto-plastic
Tiedbreak (normal and shear failure) (T)	Viscoelastic

#### Validation 2: Local Brain-Skull Relative Displacement

Most published FE rat brain models were only validated against the DCD experiment. A FE rat head model reported recently by Antona-Makoshi was validated against the brain-skull relative displacement from a sagittal rotation experiment (Davidsson and Risling, 2011; Antona-Makoshi et al., 2014). Briefly, the rat was placed on a flat plane and exposed to a backward rotational acceleration. In Antona-Makoshi’s test, a hole was drilled through the rat skull at 3.5 mm posterior and 2.2 mm laterally to the right side of the Bregma. The skull cap was redesigned with a 0.5 mm diameter steel pin mounted. Then the pin was inserted into brain through the skull hole. The cap and skull were fixed with glue (Super-Bond Cand B; Sun Medical Co., Shiga, Japan). After loading, a scar was produced by the pin in the cortex due to the brain movement with respect to the skull. The scar length in the brain at the depth of 0.5, 1, 1.5, and 2 mm sections from the cortex surface were measured after the test using a 40× lens microscope.

To validate the model against brain-skull relative displacement, a finely meshed pin object as used in the test was meshed and incorporated into the FE rat head model. A 2.5 ms head rotational acceleration curve from the experiment (Antona-Makoshi et al., 2014) was applied to the model and simulation was extended to 5 ms after loading was stopped to capture lagged brain motion. The contact between the brain tissue and pin was defined as node to surface contact type (CONTACT_AUTOMATIC_NODES_TO_SURFACE).

#### Validation 3: Rat Head Kinematics Validation

**FIGURE 3:**
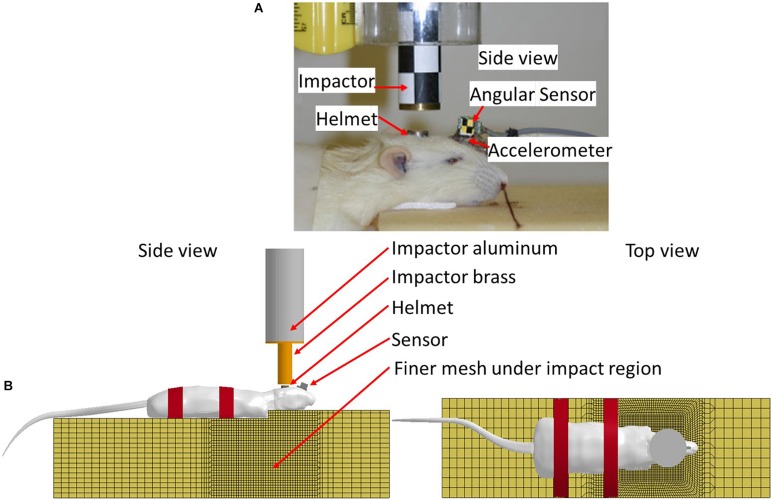
**(A)** Rat head impact experimental setup and the sensors used; **(B)** FE model set up simulating rat head impact acceleration experiment (side and top view).

*In vivo* rat head impact experiments using a modified Marmarou’s IA model reported previously were simulated (Li et al., 2011b) to validate the FE rat model. Briefly, in the experiment, the SD rat was placed in prone position on a foam bed with the head on the upper step of the foam (10 mm rise). A rat skull was exposed by a middle incision to the flesh. A titanium helmet disk (diameter: 10 mm, height: 7.5 mm, mass: 2.8 g) was attached to the exposed skull using cyanoacrylate (Elmer’s Products, Columbus, OH, United States) between the Bregma and Lambda. A brass impactor weighting 450 g was freely dropped from the two heights, 2.25 m and 1.25 m to the rat head via the helmet to induce TBI (Figure 3).

The FE models of the helmet disk, brass impactor, and foam bed were developed with actual geometry, and integrated with the FE rat full body model. The foam elements under the impact zone were finely meshed at 2 mm to accurately simulate the rat head-foam contact response and large deformation in that region. The rest of the foam was meshed with 10 mm resolution. The FE models of the rat helmet, impactor-brass component, and impactor-aluminum component were developed conforming to the geometry of the structures used in the animal experiment (Li et al., 2011b) and assigned material properties (MAT_ELASTIC) of the titanium, brass, and aluminum, respectively. The sensors attached to the skull were also meshed with the actual size and location as used in the experiment to export the head kinematics (linear acceleration and rotational velocity). The tied contact (CONTACT_SURFACE_TO_SURFACE) was defined between the helmet and rat skull, and between the sensor and rat skull/facial bone. The sliding contact (CONTACT_AUTOMATIC_SURFACE_TO_SURFACE) was defined between the impactor and helmet, and between the rat and foam. The mesh of the FE foam model in the area where the rat head being compressed into the foam upon impact was meshed with finer elements to accurately capture the large deformation (Figure 3B). MAT_FU_CHANG_FOAM (MAT_83) material model was applied to simulate loading rate dependent properties of the foam (Density: 1.362e-8 kg/mm^3^) based on the experimentally measured stress-strain curves by Zhang et al. (2011). The impactor velocity before impacting on the helmet was given as 6.15 and 4.54 m/s in our previous study for the impact height of 2.25 and 1.25 m, respectively (Li et al., 2011a). The simulations of the rat impact experiments were carried out by applying the initial velocity in negative *z*-axial direction to the impactor. The initial velocity was the average impact velocities measured respectively, from 2.25 to 1.25 m height drop experiments. The impactor was constrained in *x* and *y* directions and the bottom of the foam was constrained in all directions. The linear acceleration and rotational velocity of the head, measured by the accelerometers and angular rate sensors attached to the rat head from experiments, and the head motion captured by a high-speed camera, were used to validate the response simulated by the FE model.

### Investigation of the Effect of the Helmet Angle and Skull Stiffness on Resulting Head Response

Previously we observed large variations of the axonal changes in terms of the distribution and axonal counts between the rats impacted from the same impact height (Li et al., 2011b). This phenomenon may be associated with variability of the initial condition which may present in the test setup including variabilities of the rat helmet surface angle with respect to the impactor surface and the central or non-central contact between the helmet and impactor. The phenomena may also be related to the physical properties of the impact including the size and mass between the rats and associated skull stiffness. In the current investigation, the factors related to the impactor angle and the intrinsic difference in head skull stiffness among the rats were evaluated.

**FIGURE 4:**
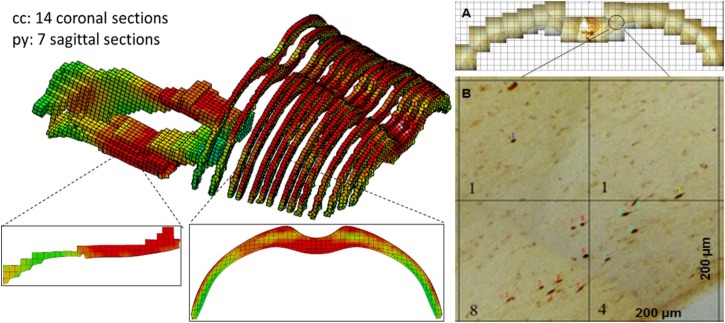
FE model sections throughout the cc and brainstem tracts with MPS contour corresponds to experiment sections of TAI counts. **(A)** One TAI coronal section of cc; **(B)** Showing an example of TAI count within four 0.2 × 0.2 mm grids from a coronal section.

The validated rat full body model was applied to simulate the effects of the variation of the initial conditions and mechanical properties and compare the resulting head kinematics and the local tissue strain distribution across the cerebral hemispheres during the impact which may explain the difference in resulting injury (Table 3). The simulation set up for contact and loading were the same as used in the validation 3. The maximum principal strain (MPS) and maximum principal strain rate (MPSR) reported in this study is based on the true strain. The MPS and MPSR were quantified for the 14 coronal sections in the cc and 7 sagittal sections in the py. These sections corresponded to the sections analyzed for axonal changes including beta-APP targeting the impaired axonal transport and RMO-14 assessing neurofilament misalignment (Figure 4).

**TABLE 3:** Simulation matrix on the effect of the initial conditions.

Variable 1: Helmet angle off 2 or 5 degrees in sagittal and lateral plane							

Case No.	Case 1	Case 2	Case 3	Case 4	Case 5	Case 6	Case 7
Impact angle variable	Helmet’s surface is in the horizontal plane	2° backward	5° backward	2° forward	5° forward	2° sideways	5° sideways
Case ID	Baseline	B_2°	B_5°	F_2°	F_5°	S_2°	S_5°

**Variable 2: Skull elastic modulus (GPa)**							

**Case No.**	**Case 1**		**Case 8**		**Case 9**		**Case 10**

Skull Young’s modulus (GPa)	15 (Baseline)		12		18		15
Varied stiffness	baseline properties		20%-Lower		20%-Higher		Skull-Rigid
Case ID	Baseline		E_skull__L		E_skull__H		E_skull__Rigid

#### Simulation of the Helmet Angle Effect

**FIGURE 5:**
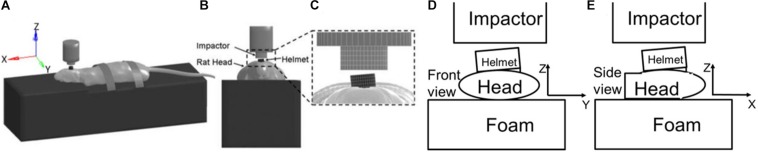
Simulation setup to test the angle effect between helmet and impactor surface. Lateral **(A)**, anterior **(B)**, and **(C)** close up views of the computer model simulation set-up for modified Marmarou rat head impact acceleration experiments, with the helmet rotated 5° to the side in the coronal plane with respect to the bottom surface of the impactor, **(D)** Diagram shows helmet rotates at 5° angle to the impactor in coronal plan, **(E)** Diagram shows the helmet rotated back angle to impactor in sagittal plane.

To test the helmet-impactor contact angle effect, the experiment of the rat head impact from an impactor falling from 2.25 m height condition was simulated. The rat head was rotated in either coronal or sagittal plane so that the helmet surface was off the horizontal plane at 2 and 5°, respectively (Figure 5). The Baseline case was the one where the helmet surface was parallel to the impactor surface in the horizontal plane. All seven cases were simulated (Table 3) and compared.

#### Simulation of the Skull Stiffness Effect

The weight of the experimental rats varied between 364 and 420 g (mean ± sd: 390 ± 13 g). The skull stiffness could be stiffer for the rat with larger mass or older age. The variation of skull properties may affect the amount of the deformation of the skull of a given rat which in turn would affect the head kinematics, and subsequently the internal brain strain response at a given impact energy. The elastic modulus of the skull defined for the FE rat head model was 15 GPa and was considered as the baseline model. In this parametric study, an additional three cases were simulated with skull stiffness varied by ±20% along with a rigid skull assuming no skull deformation under the impact (Table 3).

## Validation Results

### DCD Validation Results

**FIGURE 6:**
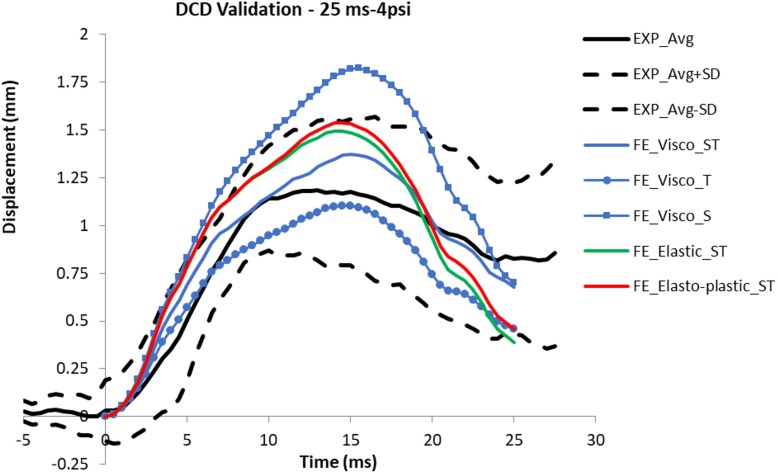
Comparison of the displacement curves predicted from the models defined with various material models and interface types with the experimental corridor (average and average ± sd).

The model with S interface resulted in a gap between the brain and skull when the brain surface was suctioned out. This gap suggested possible damages predicted at the brain/skull interface where the experiment did not observe that. The temporal profile of the displacement predicted by the S interface model did not match to the experimental curves for various cases (Figure 6). The model with T interface on the other hand resulted in much less displacement than those from ST interface. Although the average displacement histories form the T interface model with viscoelastic material fell within the average experimental corridor, the model defined with ST interface along with the viscoelastic material model matched better with displacements to most of the experiment curves in terms of temporal profiles and peak values. The displacement-time histories from the model defined with elastic or elasto-plastic meningeal layers had a faster increase following the application of the negative pressure and a faster decrease at the end of the unloading. Then, the model responses fell out of the experimental corridor at the beginning and end of the loading phases (Figure 6). The CORA (CORelation and Analysis, CORAplus V4.0.4, Developed by PDB, Partnership for Dummy Technology and Biomechanics, Germany) score for the model defined with viscoelastic meningeal and ST interface was calculated for all nine cases with an average score of 0.74 (from 0.58 to 0.92; 1: prefect match) (The validation displacement history curves and CORA scores of all 9 cases are in Supplementary Appendix Figure A1 and Supplementary Appendix Table A2, peak values in Supplementary Appendix Figure A2 section).

### Brain-Skull Relative Displacement Validation Results

The model predicted brain displacement relative to the skull at four levels beneath the brain surface was analyzed. The model results showed good match to the experimental data (Table 4). The FE model predicted brain displacement also followed the trend of the experimental data where scratch length was slightly increased at 1 mm compared to 0.5 mm beneath the brain surface, then continuously decreasing as approaching to the brain center.

**TABLE 4:** Brain-skull relative displacement in the brain: experimental and FE model results.

Depth below the cortical surface	0.5 mm	1 mm	1.5 mm	2 mm
Experiment (mean ± sd)	1.2 ± 0.12	1.2 ± 0.15	1.0 ± 0.12	0.6 ± 0.06
FE model	1.2	1.45	1.13	0.64

### Rat Head Kinematics Validation Results

The impactor displacement predicted from the FE model simulating head impact test were in good agreement with the experimental data at two drop heights, as well as the rat head linear acceleration and rotational velocity were fell within the value of experimental results, and close to the average magnitude (Table 5). The head linear acceleration and rotational velocity curves from the FE model represented typical patterns measured from experiments as reported by Li et al. (2011a, b). The comparison of the linear acceleration peak was focused on the first 1 ms duration. The rotational velocity had a lower negative peak within 2 ms and followed with a higher positive peak around 5ms, the total rotational velocity ended within 15 ms.

**TABLE 5:** FE model head kinematics validated against modified IAexperimental data.

Mean ± SEM	Linear Acc. (g)	Angular Vel. (rad/s)	Impactor Disp (mm)
Exp_2.25m (*n* = 16)/FE	855 ± 118/917.6	132 ± 11/129.5	−90.3 ± 0.5/−88.7
Exp_1.25m (*n* = 15)/FE	660 ± 44/674.3	95 ± 6/106.3	−66.2 ± 0.9/−64.0

## Parametric Study Results

### Effect on Head Kinematics

#### Helmet Angle Effect on Head Kinematics

For an ideal sagittal impact (in *x*-*z* plane), the head linear acceleration in *y*-direction and angular velocity about *x*- and *z*- axis could be ignored. In case of the baseline model representing a perfect sagittal impact, the model-predicted peak linear head acceleration at 0.4 ms occurred when the impactor impacted the helmet. For head rotational kinematics, the rotational-*y* velocity peaked at 0.8 ms when the head was in extension and was followed by the second peak at 4.5 ms in flexion. The effect of the helmet angle on the resulting head kinematics is shown Table 6. The linear acceleration was reduced for forward inclined helmet cases and increased for backward case. Meanwhile the rotational velocity was, however, reduced for backward case and increased for forward case. For the sideways case in which the head was moved out of the sagittal plane laterally, it was found that the sagittal motion both in terms of the linear accelerations in *x* and *z* directions, and rotational velocity in y axis was reduced.

**TABLE 6:** Kinematics changes compare to Flat case.

	B_2°	B_5°	F_2°	F_5°	S_2°	S_5°	E_skull__L	E_skull__H	E_skull__Rigid
Linear Acc	4%	8%	−8%	−31%	−2%	−11%	−2.80%	2.61%	3.85%
Wy(p)*	0%	1%	−1%	−4%	0%	0%	−0.08%	−0.49%	8.22%
Wy(n)*	−7%	−9%	7%	14%	0%	−2%	0.69%	−0.62%	7.76%

*Rotational velocity change: Wy(p): positive peak; Wy(n): negative peak.

**FIGURE 7:**
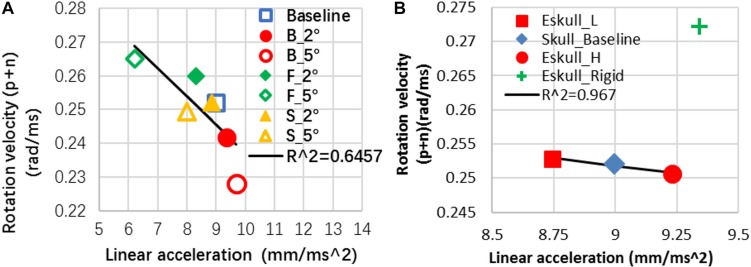
**(A)** Rat head kinematics was inversely correlated between the angular motion and linear motion in response to the helmet angle change; **(B)** head kinematics of skull effect (P.S The data point of Eskull_Rigid is excluded to show the negative relationship of other three cases in this plot).

For the cases with increased helmet angles, the patterns of change persist with increased magnitudes compare to the baseline case, except for the positive rotational velocity of sideward cases (Table 6). The first peak rotational velocity was affected more drastically by the helmet angle than the second peak. The change of the peak linear acceleration and peak rotational velocity were negatively related (Figure 7A). The head linear acceleration had 8 to −31% difference when the helmet angle varied from baseline to 5° with respect to the impactor surface (Table 6). The linear acceleration was very small in the *y* direction when helmet was flat in sagittal plane; the sideway cases significantly increased the lateral acceleration to 4.7 m/s^2^.

#### Skull Properties Effect on Head Kinematics

By decreasing the elastic modulus of the skull by 20% over the baseline value, the maximum strain in the skull was less than 0.009. This strain value did not reach the skull fracture threshold 0.01 strain set for the model. The model prediction was consistent with a low skull fracture rate (20.4% of 2.25 m cases, 0% of 1.25 m) as observed from the rat experiments. Compared to the baseline skull model, with a less stiff skull (20% less than the baseline), the head linear acceleration was reduced by 2.8%. With a stiffer skull, the linear acceleration was increased by 2.6%. For a rigid skull, the linear acceleration was increased by 3.85% with pulse time duration being 30% shorter than that of the baseline value. The change of the elastic moduli of the skull had very minimal effect (<1%) on the rotational velocity about *y* axis. The rigid skull case increased rotational *Y*-velocity by 8% (Table 6). Overall for the deformable skull, it was observed that an increase in linear acceleration usually resulted in a decrease in rotational velocity in comparison to the baseline case. The change of the peak linear acceleration and peak rotational velocity were negatively related except rigid skull case (Figure 7B). For a rigid skull absent of any skull deformation from the impact, both linear and rotational responses increased.

### Effect on Brain Response

#### Helmet Angle Effect on Brain Response

**FIGURE 8:**
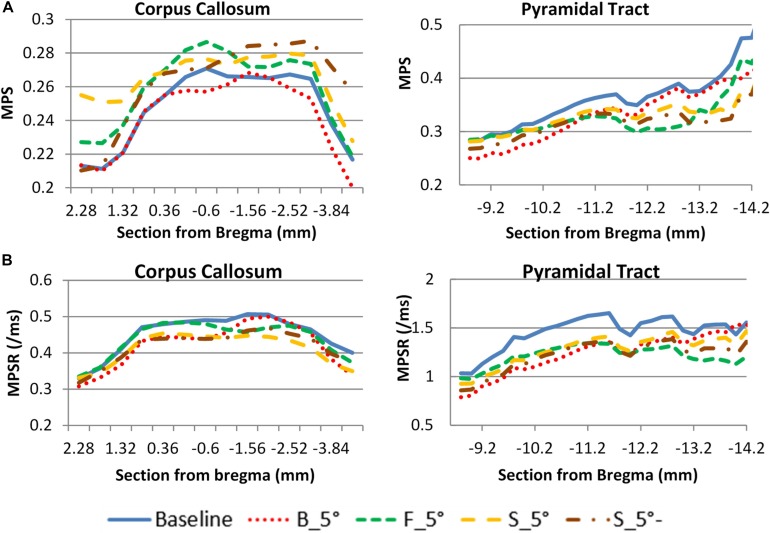
Helmet angle effect on strain comparison at two white matter structures. **(A)** maximum principal strain **(B)** maximum principal strain rate (PS. S_5°- is the MPS/MPSR response of the other side of hemisphere of S_5°case).

The head kinematics were varied as the helmet surface inclined to 5° angle. As a result, the corresponding internal brain responses were also affected (Figure 8). For the cc, the F_5°case produced a higher MPS than the other cases and was at approximately 7.3% higher than the baseline case (perfectly horizontal helmet). The B_5°case produced the lowest average MPS in the brain which was 7.6% less than the baseline case. The change in MPSR response was different than the MPS where the highest value was found in the baseline case and the lowest value was found in S_5°. For the py, the baseline case had both the highest MPS and MPSR while as the F_5° had the lowest MPS and MPSR than the other cases. The results showed that the effect on the MPS and MPSR were not only on the magnitude, but also the distribution trend as the helmet angle slightly off forward or backward in the sagittal plane. The MPS of py varied from 1 to −28% compare to baseline.

#### Skull Properties Effect on Brain Responses

**FIGURE 9:**
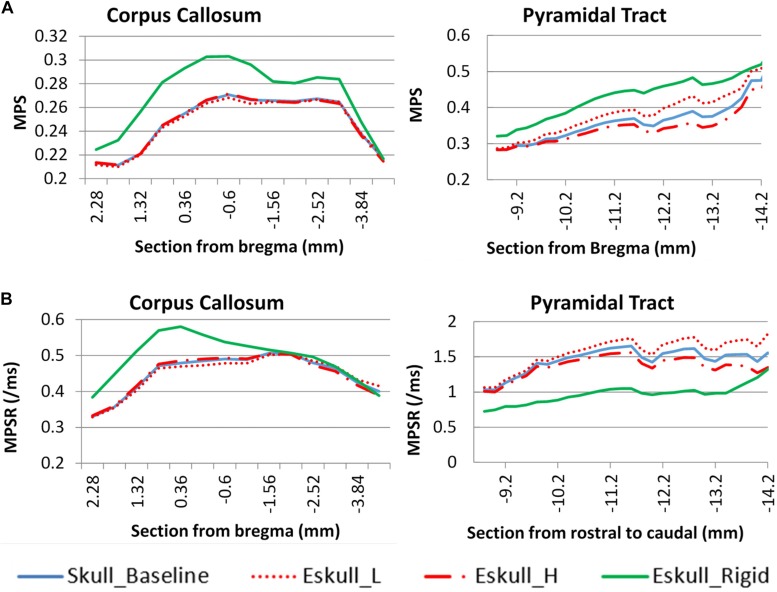
Skull stiffness effect comparison for two white matter structures. **(A)**: maximum principle strain; **(B)**: maximum principle strain rate.

As the elastic modulus of the skull varied by ± 20%, there was very small deviation of the MPS in the cc region as compared to the baseline (Figure 9). The change of MPS in the py of brain was increased with a soft skull (11%) and decreased with a stiff skull (8%) at the location −13 mm caudal to the Bregma. The MPS in the case of rigid skull, both responses of cc and py structures were dramatically increased particularly in the rostral region, however, the MPSR in the py was much lower than deformable skull cases. The change was caused by the change of head kinematics, as well as due to the increased neck stretching as compared to a deformable skull.

## Discussion

The current model was the first FE rat head model that defined transversely isotropic material properties to represent the fiber direction in major white matte tissues in the brain. The literature reported results from the material property tests suggested the behaviors of the white matter structures are transversely isotropic (Arbogast and Margulies, 1998; Prange and Margulies, 2002; Ning et al., 2006; Hrapko et al., 2008; Van Dommelen et al., 2010). This is an important step enabling the capability of the model to predict directional dependent responses to applied impact of the same energy buy varying directions in the brain such as stress/strain and related to the injury patterns. The current model was rigorously validated all available rat experimental data and showed good agreement with the experimental results.

### Model Validation

#### DCD Validation

Previous FE rat head models (Mao et al., 2006; Lamy et al., 2013) only validated the brain response in terms of the peak cortical displacement against the experimental DCD results. The current FE rat head model is the first FE model that has been validated against the peak cortical displacement values and also the temporal responses of the cortical displacement. This new validation is improved validation by verifying timing of the brain deformation in respond to a given loading. The validated model can ensure the accurate prediction of the timing of the peak deformation and the profiling resulting injury caused by the deformation.

The characteristics of the skull-brain interface was reported to be an significant factor affecting the brain displacement (Baumgartner and Willinger, 2004; Mao et al., 2006). To properly model this interface, three contact interface types available in the FE solver were investigated and the model results showed that the cortex surface displacement profile was influenced by the type of the interface. The T interface resulted in much less displacement as compared to the ST interface. It is obvious, since the T contact does not permit tangential motion occurring between the two surfaces. The S interface, on the other hand, induced a gap between the brain and skull in the subdural space. This suggested that the brain tissue moved together as it should have been deformed in shape only given that the brain tissue is a very soft material with shear modulus at the order of a few kilopascal. The prediction of gap could result in unrealistic deformation in the brain tissue.

The material properties defined for pia-arachnoid was found to play an important role as far as how this membrane could withstand the external force and the time-dependent response. The experimental study suggested that the tensile and traction properties of the pia-arachnoid in tension and traction can be modeled as elastic, elasto-plastic or viscoelastic material. In the DCD validation study, the results from the pia-arachnoid defined with three different material models showed that the viscoelastic material exhibited a delayed cortical surface deformation profile during the loading and unloading phases which matched with experimentally measured profile. In contrast, due to lacking viscous property, the displacement curve from the elastic and elasto-plastic pia-arachnoid raised and dropped much sooner and fell out of the experimental corridor. Furthermore, the rate depended viscoelastic model was able to match all the cortical deformation profiles for nine cases with a variety of loading rate. This is because the rate-dependent response can only be simulated by the viscoelastic pia-arachnoid membrane. By defining pia-arachnoid as a viscoelastic material model along with ST interface for the brain-skull interface, the current rat head model was validated against dynamic cortical deformation experimental results and demonstrated adequate biofidelity of the computer model.

#### Brain-Skull Relative Displacement Validation

The FE rat model was further validated against the measured brain displacement at 0.5, 1, 1.5, and 2 mm depth below the cortical surface from a lateral head impact experiment reported by Antona-Makoshi et al. (2014). The brain excursion predicted by the current rat head model matched well to the experimental data. For the temporal profile of the brain excursion, the model predicted peak displacement at 1.3 ms in one direction followed by the second peak at 4.3 ms in the opposite direction as the head decelerated to stop. Recently, Antona-Makoshi et al. (2014, 2015) reported their rat head model validation results based on the same set of experiments. Their results showed that the brain reached maximum displacement at 0.5 ms and later at 2 ms in the opposite direction. That the brain displaced faster in their rat brain may suggest less viscous properties defined for the brain tissue. This is confirmed by the decay constant used in that rat FE model which was about 5/s as compared to the 50/s used for the current model. Mesh size was 0.35 mm of Antona-Makoshi’s rat brain compared to current rat FE mesh was 0.2 mm. While the pin diameter was 0.5 mm, Antona-Makoshi’s rat brain had only one node in contact with the pin during simulation, but there were two nodes in the current FE model. That means the rat brain of this study could experiencing more resistance than in Antona-Makoshi’s simulation, so the current brain motion was also delayed by the pin resistance.

The validation study revealed that explicitly modeling the pin inserted in the brain as used in the experiment was an essential element to map the brain motion accurately. Without incorporating the FE mesh of the pin, the predicted brain displacement was approximately two times the experiment value. With the inclusion of the FE pin with the proper size, geometry, and most importantly the interface along with coefficient of the friction defined between the pin and the surrounding brain tissue, the rat model predicted displacement at all four depths closely matched to the experimental results.

#### Rat Head Kinematics Validation

This is the first rat FE model validated against the data from a modified Marmarou’s head IA model. This rat FE model enables simulation of head impact acceleration injury, which is the most important pre-clinical TBI model relevant to the biomechanics of axonal injury in humans. Together with the results of validation 1 and validation 2, this FE model can also predict the internal brain responses accurately under the IA loading condition which involves significant head motion in the sagittal plane.

The FE model was validated against three parameters measured from the modified IA model, including head linear acceleration, head rotational velocity, and head extrusion into the foam. The FE head kinematics curves matched quit well with the typical experimental curves. The peak values of the three parameters also showed good agreement with average experimental data. These results indicated that the FE model was capable of transferring the impact energy into the rat head in a proper way and subsequently to the supporting foam as demonstrated in the experimental setting. The reaction of foam was also properly simulated to reproduce the rat head dipping into the foam. The accurate material properties of the supporting foam assured the proper prediction of the head excursion during impact and rebound phases.

### Effects of the Helmet Angle and Skull Stiffness on Rat Head/Brain Responses

#### Head Responses

Although our modified Marmarou’s IA injury model precisely controlled the repeatability and consistency of the impact energy delivered to the animal head, the variability of the resulting rat head kinematics and severities of the axonal pathology in the major white matter tracts from the same group of rats under same impact severity were still observed (Li et al., 2011a, b). These biomechanical response variations and subsequently the brain injury variations may stem from some of the initial conditions in this experimental model that were not easily controlled, such as the plane of helmet disk may be inclined at a small angle with respect to the impacting surface of the impactor, and or the difference in rat skull stiffness associated with different head weights. By conducting parametric studies using the FE modeling, the biomechanical cause and effect relationships can be identified and evaluated may explain the severity and extent of the injuries produced by this widely used rodent impact acceleration injury model.

The results from the simulation revealed that the rat head kinematics was affected significantly (31%) by the helmet angle inclined to 5° and slightly (8%) by the skull Young’s modulus properties varied by 20%. At the baseline skull properties, the head linear acceleration was inversely correlated to the head rotational velocity as the helmet plane inclined by 5°. This implies that the overall energy transfer was similar, but the relative levels of linear and rotational kinematics components were altered which in turn could affect brain responses and resulting brain injury.

#### Brain Responses

Biomechanically, rapid changes of the head motion and the local skull deformation from a head impact induce stress and strain in the brain. By comparing the brain responses from the parametric study, the brain strain response in the cc was found to be related to the magnitude of the head kinematics which was affected by helmet plane. The strain response in the py tracts of the brain stem was influenced by changes to the head kinematics and the skull stiffness. It was also found that increased py strain was associated with increased stretching in the neck. This effect was more drastic in case of the rigid skull where the impact energy was all converted to the kinematics due to lack of the local deformation in the skull.

Compared to the strain in the cc, strain in the py was dictated by the amount of skull deformation caused by the direct impact. By varying the elastic modulus of the skull, it was observed that py strain at the caudal end increased when the skull was assigned 20% lower Young’s modulus than the baseline value. This is due to the deformed skull compressing the brain below the impact, and the incompressible brain content was pushed and moved toward the foreman magnum (the opening of the skull) due to cranial volume reduction. The movement of the brain tissue caused high principal strain in the brain stem region. On the other hand, the brain deformation in the cc (which is beneath the impact region) appeared to be less sensitive to the deformation occurring at the brain surface.

## Conclusion

A FE rat body model with a high-resolution detailed FE rat head has been developed. To our best knowledge, this is the first FE rat model that has been subjected to rigorous validations against all available biomechanical data measured from *in vivo* rat experiments. The material behavior of the pia-arachnoid and contact type at the brain/skull interface played key roles for matching both temporal and spatial profiles of the brain displacement from DCD test. The rat brain material properties had been validated against brain-skull relative displacement at various subcortical regions in response to head sagittal rotational test. The rat model has also been validated against head kinematics measured from head impact acceleration injury experiments with accurate boundary condition provided at the brainstem/spinal cord by the rat full body model.

The transversely isotropic material (MAT_92) has been incorporated in rat brain FE model at first time. It enables the simulation of the directional properties of these highly aligned white matter tracts. The model has capabilities predicting impact direction dependent responses in various white matter tracks and may improve our understanding of the biomechanical basis for various white matter injury in a variety of environment. The followings are the summary of the key findings from the current study:

• The FE models of impact head injury, once rigorously validated, can assist in understanding the underlying

• biomechanical variables responsible for the severity of insult received by the animal and subsequently the severity of injury from mechanical trauma.
• A small deviation in helmet inclination angle from 0 to 5° affects the resulting head linear acceleration by 31% and rotational velocity by 19%. This may explain large dispersion of the head kinematics (321–2313 g; 52–181 rad/s) measured from the experimental IA model even with improved consistency and repeatability in impact energy.
• The FE analysis showed that strain in the corpus callosum was affected by the head kinematics (linear and rotational), and axon bundles of the brainstem was also affected by the local skull deformation and head-neck boundary conditions. This again may explain the deviation of the axonal counts between the tests in our previous studies.
• This study revealed two biomechanical factors contributing to the variability of head and brain responses from Marmarou’s IA model. Identification of these variables may help explain the variability of injury severity observed among experiments and across different labs.

The ongoing analysis focuses on the correlation of the biomechanical response maps from the FE rat brain model to the TAI injury maps to establish tissue level thresholds for predicting white matter injury. Such tissue level threshold can be directly translated to the FE human brain model to improve its predictability for closed head brain injury.

## Data Availability Statement

The datasets analyzed in this article are not publicly available. Requests to access the datasets should be directed to lzhang@wayne.edu.

## Ethics Statement

The animal study was reviewed and approved by the Wayne State University Animal Care and Use Committee.

## Author Contributions

RZ involved in the study concept and method design, data acquisition, data analysis, interpretation of data, and drafted the manuscript. YL involved in data acquisition and analysis. JC involved in critical revision of the manuscript for important intellectual content and obtained funding (co-PI). LZ, involved in study concept and method design, data acquisition, data analysis, interpretation of data, composed the manuscript, critical revision of the manuscript for important intellectual content, obtained funding (PI), and provided supervision.

## Conflict of Interest

The authors declare that the research was conducted in the absence of any commercial or financial relationships that could be construed as a potential conflict of interest.
