# Supplementary material for: Investigate the Variations of the Head and Brain Response in a Rodent Head Impact Acceleration Model by Finite Element Modeling
Source: Front Bioeng Biotechnol. 2020 Mar 18;8:172. doi: 10.3389/fbioe.2020.00172 (PMC7093345; doi:10.3389/fbioe.2020.00172)
Supplement: Supplementary file 1 [file Table_1.DOCX]

Appendix table:

Table A1: Material property of white matter structures define for transversely isotropic material models

|  | Hyperelastic constants (kPa) | | Fiber pre stretch  constant | | Fiber stiffness (kPa) | Shear relaxation moduli | | Characteristic time (ms) | |
| --- | --- | --- | --- | --- | --- | --- | --- | --- | --- |
|  | C1 | C2 | C3 | C4 | C5 | S1 | S2 | T1 | T2 |
| CC | 0.177 | 0 | 80 | 46 | 1.42 | 0.8973 | 0.0741 | 80 | 8000 |
| ml, mlf, and Py tracts | 0.354 | 0 | 80 | 46 | 1.42 | 0.8973 | 0.0741 | 80 | 8000 |
| Brainstem | 0.354 | 0 | 80 | 46 | 1.42 | 0.8973 | 0.0741 | 80 | 8000 |


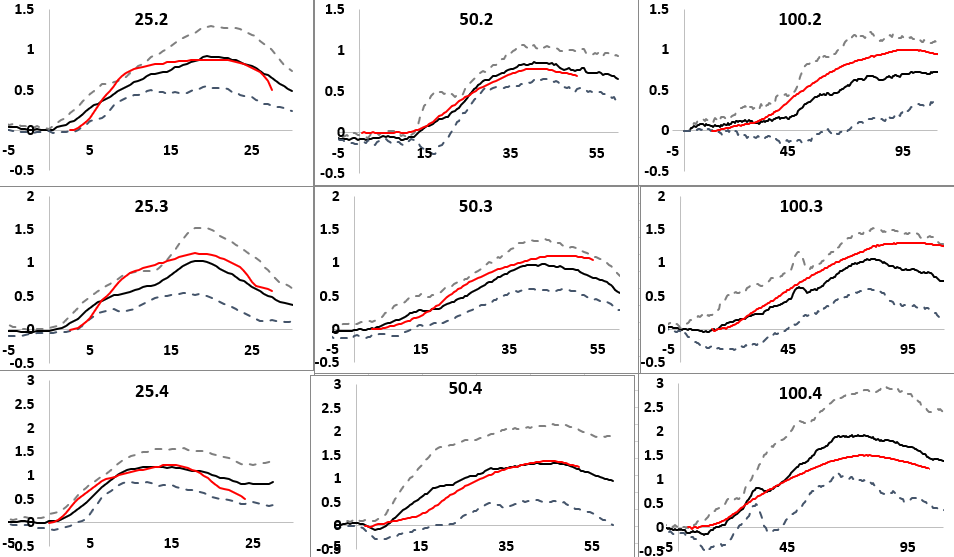


Time (ms)

Displacement (mm)

Figure A1: DCD validation displacement time history curves of all 9 cases (25ms-2psi, 50ms-2psi, 100ms-2psi; 25ms-3psi, 50ms-3psi, 100ms-3psi; 25ms-4psi, 50ms-4psi, 100ms-4psi)

Table A2 DCD validation CORA score of all 9 cases

| Cases | | Corridor | Cross-Correlation | | | Weighted Score |
| --- | --- | --- | --- | --- | --- | --- |
| Duration (ms) | Pressure (psi) |  | Phase | Magnitude | Slope |  |
| 25 | 2 | 0.8 | 0.2 | 0.8 | 0.6 | 0.66 |
|  | 3 | 0.6 | 1.0 | 0.8 | 0.7 | 0.75 |
|  | 4 | 0.8 | 0.9 | 0.9 | 0.7 | 0.84 |
| 50 | 2 | 0.9 | 1.0 | 0.9 | 0.8 | 0.92 |
|  | 3 | 0.6 | 0.9 | 0.8 | 0.7 | 0.74 |
|  | 4 | 0.8 | 0.2 | 0.9 | 0.8 | 0.69 |
| 100 | 2 | 0.5 | 0.6 | 0.7 | 0.7 | 0.58 |
|  | 3 | 0.7 | 0.9 | 0.7 | 0.7 | 0.71 |
|  | 4 | 0.7 | 1.0 | 0.7 | 0.7 | 0.77 |

Figure A2: Validation results: displacement peak values


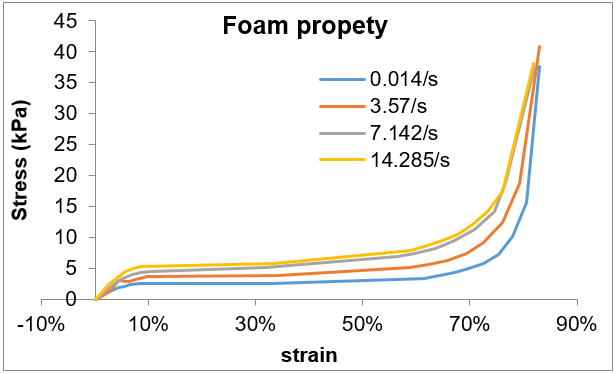


Figure A3 Foam properties at different loading rates (Zhang et al., 2011)

Abbreviation:

Diffuse Axonal Injury (DAI)

Impact Acceleration (IA)

Finite Element (FE)

Traumatic Brain Injury (TBI)

Traumatic Axonal Injury (TAI)

Corpus Callosum (cc)

Impaired Axoplasmic Transport (IAT)

Neurofilament Compaction (NFC)

Cerebrospinal Fluid (CSF)

Magnetic Resonance Imaging (MRI)

Micro Computerized Tomography (microCT)

Sprague Dawley (SD)

Pyramidal tract (py)

Trigeminothalamic tract (tth)

Medial Lemniscus Fasciculus (mlf)

Global Human Body Models Consortium (GHBMC)

Dynamic Cortical Deformation (DCD)

Maximum Principal Strain (MPS)

Maximum Principal Strain Rate (MPSR)

Standard Deviation (sd)

CORelation and Analysis (CORA)
